# Rapid and Reliable Detection of *Echinococcus multilocularis* from Faeces Using Droplet Digital PCR

**DOI:** 10.1007/s11686-020-00325-9

**Published:** 2020-12-21

**Authors:** Fabian Bagó, Franz Hoelzl, Felix Knauer, Anna Kübber-Heiss, Steve Smith

**Affiliations:** 1 Research Institute of Wildlife Ecology, Department for Interdisciplinary Life Sciences, Vetmeduni Vienna, Savoyenstraße 1, 1160 Vienna, Austria; 2 Konrad Lorenz Institute of Ethology, Department for Interdisciplinary Life Sciences, Vetmeduni Vienna, Savoyenstraße 1, 1160 Vienna, Austria; 3 Conservation Medicine Unit, Research Institute of Wildlife Ecology, Department for Interdisciplinary Life Sciences, Vetmeduni Vienna, Savoyenstraße 1, 1160 Vienna, Austria

**Keywords:** *Echinococcus multilocularis*, Magnetic capture extraction, Droplet digital PCR, Foxes, Golden jackals

## Abstract

**Purpose:**

Alveolar echinococcosis is a severe helminthic disease in humans caused by larvae of the fox tapeworm *Echinococcus multilocularis.* Austria is considered an endemic area with hotspots having up to 45% prevalence (Bagó et al. in Proceedings of the Zoo and Wildlife Health Conference 2019, Berlin, p. 91, 2019). At our facility, we have registered a notifiable increase of animals submitted for the diagnosis of *E. multilocularis* since 2016. Therefore, we investigated high throughput diagnostic methods to provide rapid and reliable results in comparison with our current method.

**Methods:**

We have developed and compared a novel method of detection using droplet digital PCR (ddPCR) combined with previous target specific extraction according to Maas et al. (Vet Parasitol 230:20–24, 2016), with our current macroscopic method “Shaking in a Vessel Technique” (SVT) by Duscher et al. (Parasitol Res 95(1):40–42, 2005). We investigated 77 wild canids (72 red foxes, 5 golden jackals) using both methods. The data were analyzed using a non-Bayesian approach, applying bootstrapping to create confidentiality intervals.

**Results:**

Sensitivity for droplet digital PCR was 90.51% with the 95% credibility interval ranging from 82.50 to 96.92%, whereas mean sensitivity for SVT was 92.04% with a 95% credibility interval ranging from 84.75% to 98.36%. Additionally, a non-linear regression similar to *R*^2^ could be pointed out between the counted worms and the results gathered from ddPCR.

**Conclusion:**

Magnetic capture extraction followed by ddPCR shows strong potential as a high throughput method for diagnosing *E. multilocularis* prevalence in diverse canid populations as well as infection intensities of individual animals, giving valuable epidemiological insights of the distribution amongst wild canids as an alternative to conventional qPCR or macroscopic methods.

## Introduction

*Echinococcus multilocularis*, (LEUCKART, 1863 and VOGEL, 1955), is a zoonotic tapeworm causing the severe helminthic disease alveolar echinococcosis (AE) in humans. AE is considered a neglected zoonotic disease by the World Health Organization (WHO) [25] and the most dangerous parasitic disease for humans living in the northern hemisphere. Various canid species can serve as final hosts, including red foxes (*Vulpes vulpes*, LINNAEUS, 1758), arctic foxes (*Vulpes lagopus*, LINNAEUS, 1758), golden jackals (*Canis aureus*, LINNAEUS, 1758), racoon dogs (*Nyctereutes procyonoides,* GRAY, 1834), wolves (*Canis lupus*, LINNAEUS, 1758) and domestic dogs (*Canis lupus familiaris,* LINNAEUS, 1758). Occasionally, even domestic cats (*Felis catus,* LINNAEUS, 1758) have been found to serve as final hosts of the parasite [11]. As intermediate hosts in Austria there are various small rodent species that have been identified including voles [7]. Even larger rodents such as the European beaver (*Castor fiber*, Linnaeus 1758*)* have been reported to serve as intermediate host of *E. multilocularis* in Austria [18]. Austria is considered an area with a generally high prevalence of *E. multilocularis* among red foxes (*Vulpes vulpes*) with regional prevalences in the past ranging from 0 to 35% but only a few cases of human AE, which may be due to underreporting [2, 10, 20]. More recent data from our institute showed local hotspots with prevalences as high as 45% in red foxes [1]. It is known that intensive rabies eradication programs through bait vaccines and the change of society´s behavior towards foxes, have increased fox populations in Europe, including Austria [4, 8]. Due to the increase of public and stakeholder (i.e., authorities, hunters) interest, as well as the rising demand for testing animals ante mortem, we investigated new methods for the detection of *E. multilocularis*. Techniques based on floatation principles are potentially very cheap and efficient but as wild canids provide habitat for various other parasites from the family of *Taenidae* (LUDWIG, 1886) it is not possible to distinguish between the individual taxa within the group [13]. Shaking in a vessel technique (SVT) is a modified sedimentation method for detecting various gastrointestinal parasites post mortem [2, 3]. At the Research Institute of Wildlife Ecology, it has been used since 2014 on over 400 samples from foxes (*Vulpes vulpes)*, golden jackals (*Canis aureus*) and wolves (*Canis lupus*) for routine diagnostics with a focus on the detection of potentially zoonotic parasites like *E. multilocularis* and *Alaria alata* (GOEZE, 1782)*.* While the SVT has proved its worth in terms of reliability and sensitivity, it is labour intensive and has only low throughput.

The aim of our study was to find and establish a high throughput method having a comparable reliability to our current method, the SVT. Additionally, we were interested in the possibility to quantify the burden of worms in individual animals. An increasing number of studies suggest the use of target specific capture using biotinylated paramagnetic beads to improve DNA extraction success from canid faeces [15, 17, 22]. Besides extraction methods using biotinylated beads there are multiple commercial stool extraction kits available as well as amplification techniques using nested PCR, which have been used by various authors to detect *E. multilocularis,* i.e., by Karamon et al. [11]. We developed a DNA-based protocol incorporating the extraction method of Maas et al. [15] and combined it with the high sensitivity and reliability of droplet digital PCR (ddPCR) for semi-quantitative detection. Droplet digital PCR (ddPCR) is a relatively new method providing repeatable, quantifiable and comparable results [21]. It is capable of avoiding problems from inhibitory factors by separating a sample in up to 20,000 individual PCR reactions in a single well through the creation of small emulsion oil droplets.

## Materials and Methods

### Sampling

In total, 77 samples were collected from two canid species. From December 2018 until January 2020 we sampled 72 red foxes (*Vulpes vulpes*) as well as 5 golden jackals *(Canis aureus)* during routine necropsies. The animals were shot, or found dead during regular hunting activities and dissected at the wildlife pathology facility of the Research Institute of Wildlife and Ecology in Vienna. No animal was shot purely for research purposes. During necropsy, the whole lower gastrointestinal tract consisting of stomach, small intestine, large intestine and rectum was removed from the carcass and immediately sealed into sterile, food safe, polyethylene bags using a foil heat sealer (^®^Polystar 413 m, Hamburg) to reduce risk of staff infection. Necropsy was performed in a certified necropsy room with adequate air cleaning devices by veterinary-pathologists and all the staff in the necropsy hall wearing personal protective equipment according to the laboratory´s standard operating procedure for the dissection of free ranging canids. After dissection, the bagged and sealed intestines were frozen at − 80 °C for at least five, but usually 14 days as recommended by the WHO and Duscher et al. [5] to inactivate zoonotic pathogens, especially worms and eggs of *E. multilocularis* and thus minimizing health risks during handling. Before starting the diagnostic procedures, samples were thawed in water baths at room temperature. Each sealed intestine was transferred to a sterile plastic tray where the bag was cut open and emptied using sterile surgical instruments to ensure no cross-contamination among samples. Then an incision on the rectum was made to transfer approximately one gram of faeces into a 15 ml plastic tube (®VWR). The tubes were weighed on a digital precision-scale (®Kern PCB 8000-1, Balingen) to ensure that enough material was taken. Differing values from ± 0.05 g were tolerated, if greater differences were registered faeces were added or removed. The tubes were labelled using water- and heat-proof stickers to prevent ID loss during the DNA extraction process. Until further processing, the tubed samples were frozen again at − 20 °C.

### Shaking in a Vessel Technique (SVT)

SVT has a declared sensitivity of 96.2% [3] and we use it as an alternative to the “golden standard”, the Sedimentation and Counting Technique (SCT) by Eckert [6] as suggested by Duscher et al. [3]. In addition to showing high sensitivity in low prevalence areas [3], SVT provides a valuable insight into the burden of parasites in animals investigated, an important component when monitoring the overall health status of free ranging canid populations.

After the intestines were freed from mesentery, the stomach and the large bowel were removed. Then the intestine was inserted into a 1 l plastic vessel with a removable lid having a 500 µm wide mesh sealed inside. Water was added and afterwards removed from the vessel via shaking. This process was repeated multiple times until clear fluid flowed from the vessel. The lid was opened, and the small bowel was removed from the jar. During the removal, the small intestine was scraped off using two sterile surgical tweezers. This was followed by another step of washing and shaking, providing a small amount of liquid containing all intestinal content larger than 500 µm. The liquid was transferred to another sterile plastic vessel, labelled with the sample number to await further processing. The sample was investigated in small fractions of 50 ml in a glass petri-dish having a black spiral on the bottom under a stereomicroscope at 8–80 × magnification (®Olympus). Adult worms of *E. multilocularis* were counted, whereas other visible parasites were noted down using a semi quantitative scale (1–9 parasites =  +; 10–100 =  ++; 100–100,000 =  +++).

### Droplet Digital PCR (ddPCR)

#### Target Specific DNA-Extraction

Extraction was carried out strictly according to the protocol provided by Maas et al. [15] with a starting quantity of one gram of faeces. Magnetic capture-based DNA extraction from faeces was conducted using capture oligos CapF and CapR targeting the 12S ribosomal RNA of *E. multilocularis* as described in the manuscript of Maas et al. [15]. DNA sequences for primers, oligos and probe are detailed in Table 1. The extracted DNA was frozen at − 20 °C in the final elution medium of 100 µl milliQ-RNAse free water until further processing.

**Table 1:** Genetic sequences used in the protocol

Name	Sequence	References
CapF	GTTATGGTGT TGTTATAAAT AAATTTTGTT AAGATATATG TGGTACAGGA TTAGATACCC CATTAA	[15]
CapR	TCAGTAATAA CCGAGGGTGA CGGGCGGTGT GTACCTGAGC TAAACTCAAT TCACATCAAC AAACTAAT	[15]
Em3	ATATTACAAC AATATTCCTA TC	[15]
Em4	ATATTTTGTA AGGTTGTTCT A	[15]
Probe EM_MGB	FAM_TAGTAA[T]GTAA[G]TTTG[T]GTAGT-BHQ1	Probe sequence based on the sequence by Maas et al. [15]

#### Droplet Digital PCR

For ddPCR, we used a minor groove binding probe (Probe EM_MGB) and internal primers Em3 and Em4, based on the primers and probes by Maas et al. [15] ordered from ®Eurofins Genomics, Ebersberg. The master mix for ddPCR consisted of 10 µl Supermix for Probes (No dUTP) (^®^BioRad, USA); 18 pmol (10 pmol/µl) primers Em3 and Em4 each; 5 pmol (10 pmol/µl) Probe EM_MGB; 0.3 µl bovine serum albumin (BSA); 2.2 µl RNAse free H20 (^®^Qiagen, Hilden); and 3.4 µl of sample DNA with a total reaction volume of 20 µl. Droplets were created using a QX200 Droplet Generator (^®^Biorad, USA). Samples were amplified in a 96-well Thermocycler (BioMetra TOne 96G ^®^Analytik Jena). Starting with initial hotstart for 10 min at 95 °C, followed by 55 cycles of 95 °C with 30 s hold and 51 °C with 2-min hold. After the 55 cycles, there was a 10 min step at 98 °C to stabilize the oil droplets. To limit the phenomenon of “droplet-rain”, which can inhibit the proper interpretation of results the ramp rate was set to 1 °C/s at all steps of the reaction [23]. Additionally hold temperatures were changed from 55 °C with the original method by Maas et al. [15] to 51 °C as internal testing through gradient PCR revealed better amplification results when lowering the temperature. Sample analysis was carried out using a QX200 Droplet Reader (^®^Biorad, USA) and the software QuantaSoft (^®^Biorad, USA). When the software did not detect a call of positive droplets automatically, the threshold was manually set directly above all unamplified droplets as determined from the negative control reaction. Samples with just a single positive droplet, were repeated and then counted as positive if detected again in the replicate. There was no evidence of systematic inhibition as described by Maas et al. [15] and Isaksson et al. [9] throughout the ddPCR step. Although negative samples could potentially be a result of PCR failure, this effect is likely to have been largely eliminated due to the dilution effect across the large amount of individual reactions (droplets) per sample volume in a single run. Nevertheless, possible inhibition during the PCR step of the extraction process cannot be ruled out, although the validation of Maas et al. [15] suggests this potential drawback is minimal.

### Statistical Data Analysis

Data analysis was carried out using the open source software R [19]. We decided against a Bayesian approach used in similar studies (see discussion), and rather opted for an approach with applied classical statistics. For both, the SVT and ddPCR with previous magnetic capture extraction, the assumption of the model is that specificity is set to 1 reflecting the low likelihood of false positives. Although, in practice, misidentification of *E. multilocularis* is a remote possibility, the extreme low level of occurrence does not substantially affect the model. If the data revealed very low concentrations of DNA, the sample was amplified in a second run to check repeatability. The partitioning of each reaction into thousands of droplets ensures independent and direct quantification creating high confidence in any positive signal [21]. As the combination of data from both methods provides comprehensive information about the individual infection status from *E. multilocularis,* this combined dataset was used as the reference to compare both individual methods to the overall reference values. From that we derived sensitivity for each individual method using 10 000 bootstrap iterations to create 95% credibility intervals, relative to the suspected “true” status of infestation. To estimate the correlation between the PCR results and the counted worm numbers an additive regression model was used (package mgcv, by Wood [24]).

## Results

### Test Results

Using SVT 58 out of 77 (75.32%) samples were tested positive, all of which came from foxes. From the 77 samples tested with magnetic capture extraction following ddPCR 57 (74.03%) were positive (56 from foxes, 1 from golden jackal). The total agreement between methods was 66 out of 77 samples (85.71%). Regarding the samples positive for *E. multilocularis* there was an overlap of 52 out of 58 samples (89.66%), but we have also recognized different outcomes of both tests (Table 2). The combined data detected a total of 63 positive samples out of 77 (81.82%). In terms of host species, one out of five (20%) of the golden jackals tested positive with ddPCR and zero with SVT. From 72 foxes tested, 56 individuals (77.78%) showed positive ddPCR results, whereas 57 (79.17%) were positive with SVT.

**Table 2:** Absolute results from both methods tested

SVT+ and ddPCR +	SVT+ and ddPCR−	SVT− and ddPCR+	SVT− and ddPCR−
52	6	5	14

### Results from the Statistical Data Analysis

Mean sensitivity for SVT was 0.9204 with a 95% credibility interval ranging from 0.8475 to 0.9836. For ddPCR, mean sensitivity was 0.9051 with the 95% credibility interval ranging from 0.8254 to 0.9692. Data are summarised in Table 3.

**Table 3:** Calculated sensitivities from a bootstrap using 10,000 iterations for both individual methods after setting the combination of both as reference

Sensitivity	Median	95% Credibility interval
SVT	0.9204	0.8475–0.9836
ddPCR	0.9051	0.8254–0.9692

**Fig. 1:**
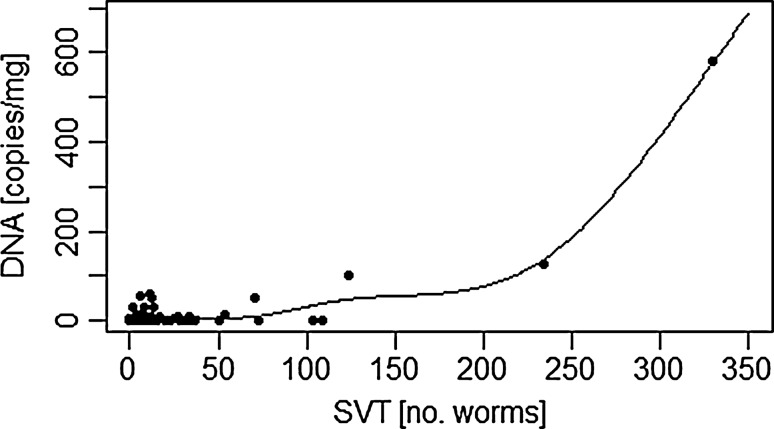
Relationship between the quantitative results of SVT and DNA concentration of copies per milligram using an additive model with a non-parametric spline (deviance explained: 95%, R package mgcv)

The comparison of the two methods via regression analysis shows a non-linear regression similar to *R*^2^. In Fig. 1 the data displays a regressional correlation between the actual worm burden of counted worms with SVT and the measured copies of DNA strains per gram faeces received from the PCR.

## Discussion

We aimed to develop and establish a method suitable of supplementing or replacing our current diagnostic method for detecting *E. multilocularis*, SVT. The comparison of both methods in our study revealed almost equal levels of sensitivity with acceptably small credibility intervals, indicating that target capture extraction and ddPCR provides a viable alternative to SVT. Somewhat surprisingly the published sensitivity of 96.2% by Duscher et al. [3] was not achieved in our study (although the point estimate did lie within our 95% CI). This could be explained by the limited sample size of our study on the one hand, or on the other hand may be caused by stochastic factors inhibiting the interpretation of SVT which have not been published before. We suspect that nutritional debris in the intestines (e.g., piles of hair, semi-digested fruit, bones, semi-digested dog/cat food) can impede the visual search for adult *E. multilocularis*. This could potentially be solved by adding an additional sieving step, but the possibility remains that the worms would adhere to digested content and not enter the shaking vessel. Our results from ddPCR suggest promisingly higher sensitivities than previously published essays using qPCR. Further studies calculating the analytical sensitivity of the assays are needed to deliver truly comparable results. The likely explanation for the apparent higher sensitivity could be the use of ddPCR instead of conventional qPCR. As ddPCR dilutes DNA extracts across 20,000 individual reactions per sample in a single well, it delivers better performance and less inhibition during PCR detection, even with challenging substrates such as faeces. Another co-factor for our increased sensitivity could have been that due to multiple freeze-thawing cycles, normally impervious egg membranes were disrupted and made target-specific capturing more efficient for the oligonucleotides [12]. This is potentially a coincidental discovery as samples were frozen multiple times for logistic reasons. Further controlled testing will confirm if multiple freeze-thaws has a general benefit and can be incorporated into the standard protocol. The common limitation of every PCR-based approach revolves around the efficient extraction of inhibitor-free DNA. For faecal derived DNA, this is always a challenge, but the use of target specific capture is a step towards minimizing this effect below clinical detection thresholds.

Numbers of cases to be investigated have continued to rise throughout Europe, as the numerous novel molecular approaches for the detection of *E.multilocularis* can be found in the literature. Furthermore, it can be assumed that public interest among stakeholders (i.e. hunters, authorities, environmentalists) is growing equally with no limitations to a single country only. SVT allows a sample throughput of approximately 40–50 samples per week, depending on whether a quantification of worms has to be performed, whereas molecular assays allow a much higher sample throughput. Depending on the capacity and availability of technical devices needed to perform the assay, at least 96 samples can be processed per week with the potential to scale up according to automation possibilities. The practical steps of the assay do not require advanced knowledge on the morphology of *Echinococcus spp.,* so all technical staff in the laboratory could perform target specific extraction after a few supervised rounds of extraction. Alternatively, for SVT to be sensitive and specific it is important to have experienced, well trained investigators which clearly requires much time and effort. Apart from sample throughput, a major advantage of ddPCR is that only faecal material is needed for processing. Recently deceased and living animals (domestic and wild) can be tested, giving insights into the distribution of the parasite amongst possible new local sylvatic hosts like wolves (*Canis lupus).* As these animals cannot be hunted in most European countries, scats are often collected and analyzed genetically as part of national monitoring programs. The same scat can then be used for magnetic capture extraction and ddPCR to monitor the presence/absence of *E. multilocularis.*

Previous studies have used bayesian models based on latent class analysis (LCA) [15, 22]. Our data showed an overwhelming dependence on the informative priors needed in LCA and would not represent an objective result, so an approach using classical statistics was chosen. To improve comparability with other molecular methods, further studies are needed to minimize the dependence of priors derived from SVT, and thus allow Bayesian models (e.g., LCA) to be applied.

Although our results are very encouraging, several limitations of the molecular approach remain to be addressed. A problem that can occur with molecular methods results from the stochastic nature of DNA extraction from a single subsample of degraded faecal matter [15, 22]. The probability of false negative results decreases with the amount of sample taken from a scat or the rectum. Single point sampling of just a gram of faecal material runs the risk of missing the portion of stool containing worms or eggs. To resolve this problem, it is suggested to perform additional pre-extraction steps and pooling for homogenization before DNA capture [16]. In our study, we did not explore multiple pre-extraction steps as within the protocol but a method of bead beating as suggested by Maksimov et al. [16], could further increase the sensitivity of our assay. Nevertheless, a molecular assay will not be able to deliver an equivalent quantification of total worm burden as seen with macroscopic methods such as SVT as the amount of material tested is completely different. As previously mentioned, correlations could be improved by implementing pre-extraction steps such as homogenization or concentration of DNA from the faecal matter. Another limitation of molecular methods is the inability to distinguish between a patent infection or the mere presence of exogenous DNA in the sample from the consumption of an infested intermediate host. This problem could be overcome by testing a host multiple times, but with wildlife that is rarely possible. Finally, samples may be so degraded from environmental exposure that no eggs/parts of worms remain in the faeces and DNA can no longer be detected.

## Conclusion

Due to effects of urbanization, more human settlements are encroaching upon wildlife habitats and wildlife are likewise more prevalent in urban areas. It has been suggested that the urban spread of Echinococcosis is determined by various factors such as the structure of urban environments [14]. As hunting is prohibited or controversial in many of urban areas in Europe, molecular assays can provide a non-invasive method to conduct epidemiological studies by analyzing host-scats in urban areas. Diagnosing *E. multilocularis* has never been easy or quick and presents substantial challenges in terms of staff safety. Freezing of intestinal tracts or even whole foxes requires large amounts of freezer space which is at a premium in most research labs. Magnetic capture extraction followed by ddPCR can be used as an alternative, sensitive molecular method for the diagnosis of *E. multilocularis* requiring less freezing capacity and allowing higher sample throughput and the possibility for in vivo testing of canids. When information on the presence of the parasite is needed and estimation of infection intensity is important, ddPCR could be used instead of traditional macroscopic techniques. Especially with rising requests for investigation and public interest growing in testing domestic animals, our method can deliver sufficient, fast, and repeatable results for the detection of *E. multilocularis.*

## Data Availability

Data are available upon reasonable demand at the corresponding author.

## Code availability

Code is available upon reasonable demand at the corresponding author.
